# Genome- and epigenome-wide association studies identify susceptibility of CpG sites and regions for metabolic syndrome in a Korean population

**DOI:** 10.1186/s13148-024-01671-5

**Published:** 2024-04-29

**Authors:** Ho-Sun Lee, Boram Kim, Taesung Park

**Affiliations:** 1 Forensic Toxicology Division, Daegu Institute, National Forensic Service, Chilgok-gun, 39872 Gyeongsangbuk-do Korea; 2 Interdisciplinary Program in Bioinformatics, Seoul National University, Seoul, 08826 Korea; 3 Department of Statistics, Seoul National University, Seoul, 08826 Korea

**Keywords:** Metabolic syndrome, Epigenome-wide association study, Genome-wide association study, TXNIP

## Abstract

**Background:**

While multiple studies have investigated the relationship between metabolic syndrome (MetS) and its related traits (fasting glucose, triglyceride, HDL cholesterol, blood pressure, waist circumference) and DNA methylation, our understanding of the epigenetic mechanisms in MetS remains limited. Therefore, we performed an epigenome-wide meta-analysis of blood DNA methylation to identify differentially methylated probes (DMPs) and differentially methylated regions (DMRs) associated with MetS and its components using two independent cohorts comprising a total of 2,334 participants. We also investigated the specific genetic effects on DNA methylation, identified methylation quantitative trait loci (meQTLs) through genome-wide association studies and further utilized Mendelian randomization (MR) to assess how these meQTLs subsequently influence MetS status.

**Results:**

We identified 40 DMPs and 27 DMRs that are significantly associated with MetS. In addition, we identified many novel DMPs and DMRs underlying inflammatory and steroid hormonal processes. The most significant associations were observed in 3 DMPs (cg19693031, cg26974062, cg02988288) and a DMR (chr1:145440444–145441553) at the *TXNIP*, which are involved in lipid metabolism. These CpG sites were identified as coregulators of DNA methylation in MetS, TG and FAG levels. We identified a total of 144 cis-meQTLs, out of which only 13 were found to be associated with DMPs for MetS. Among these, we confirmed the identified causal mediators of genetic effects at CpG sites cg01881899 at *ABCG1* and cg00021659 at the *TANK* genes for MetS.

**Conclusions:**

This study observed whether specific CpGs and methylated regions act independently or are influenced by genetic effects for MetS and its components in the Korean population. These associations between the identified DNA methylation and MetS, along with its individual components, may serve as promising targets for the development of preventive interventions for MetS.

**Supplementary Information:**

The online version contains supplementary material available at 10.1186/s13148-024-01671-5.

## Background

Metabolic syndrome (MetS) is considered a representative example of complex traits, as it is defined by a combination of traits, including central obesity, insulin resistance, dyslipidemia, and hypertension. Therefore, it is important to identify the causal interplay between MetS and its associated risk factors. Previous epigenome-wide association studies (EWAS) have emphasized the epigenetic role of lipid metabolism as a major contributor to the pathogenesis of MetS and its phenotypic outcomes. [1, 2] Both obesity [3, 4] and hypertension [5], as components of MetS, have also been reported to be regulated by epigenetic mechanisms. Recently, various EWAS conducted among European, African American, and East Asian populations have identified certain CpG sites near *TXNIP, ABCG1, SREBF1, IGF2BP1 and GFPT2* that are associated with MetS [6–8]. While numerous studies have revealed methylation sites linked to MetS, the causal relationship between these epigenetic modifications and the onset of the disease remains an area requiring more extensive investigation. These considerations are encountered in the context of identifying causality with other phenotypes [9] or combined effects of multiple target sites [10]. Moreover, despite numerous EWAS on MetS, identifying causal effects in MetS remains challenging compared to other metabolic disorders, such as type 2 diabetes (T2D) and cardiovascular diseases.

Research interest in genetic impacts on DNA methylation variation is especially relevant in the context of methylome changes observed in diseases [11]. Genetic variation represents an additional contributor to DNA methylation in tissues, with genetic influences estimated to account for approximately 20–80% of DNA methylation variance within a given tissue [12]. Most of the genetic associations identified for MetS are related to individual components, exhibiting varying degrees of pleiotropy [13]. Multiple studies have successfully identified genetic variants associated with methylation quantitative trait loci (meQTLs), which appear to overlap with expression quantitative trait loci, thereby influencing phenotypes [14]. This implies that both DNA methylation and gene expression may exist along the interconnected pathway linking genetic variation and disease. Therefore, several studies have attempted to evaluate whether a causal relationship exists between DNA methylation measured in peripheral blood and various metabolic diseases [10, 14]. However, the current evidence of a mediated effect between meQTLs and related traits for MetS is still being uncovered.

To accumulate knowledge of the pathological mechanisms behind the condition at the methylation level, we performed a meta-analysis of EWAS using whole blood DNA methylation data generated by the EPIC microarray. This study included 2334 Korean individuals (1520 cases and 814 controls) from 2 independent cohorts to identify differentially methylated probes (DMPs) and differentially methylated regions (DMRs) associated with MetS and its components. Additionally, we performed Mendelian randomization (MR) analysis with cis-meQTLs to investigate whether the observed DNA methylation changes linked to genetic variation are causally linked to MetS.

## Methods

### Study participants

This study used data from the Korean Genome and Epidemiology Study (KoGES) Consortium, which includes multiple independent prospective cohorts that differ according to the residential areas of the participants. These cohorts include the Health Examinees (HEXA) study and the Korea Association Resource (KARE) study. The discovery study (KARE) used data from 1528 participants, which were obtained from the fifth 2-year follow-up phase of the KARE cohort in 2011–2012. For the replication stage (HEXA), the data from 822 participants from the HEXA cohort in 2004 (baseline) were used. Detailed information about the KARE and HEXA cohorts has previously been described [15]. Additional file 3: Table S1 provides a summary of the studies and relevant details used in the analysis. This study was conducted with bioresources from the National Biobank of Korea, the Korea Disease Control and Prevention Agency, Republic of Korea (KBN-2020-108). Approval for the study was obtained from the Institutional Review Board of Seoul National University (IRB No. E2209/001-001).

### Metabolic syndrome

MetS was defined according to the modified criteria of the National Cholesterol Education Program-Adult Treatment Panel III (NCEP-ATP III) with the appropriate waist circumference (WC) cutoff point for central obesity in Koreans [13]. MetS was diagnosed if individuals exhibited at least three of the following components: (1) WC ≥ 90 cm for men and ≥ 85 cm for women, (2) triglyceride (TG) level 150 mg/dL or pharmacologic treatment, (3) high-density lipoprotein (HDL) cholesterol level 40 mg/dL in men and 50 mg/dL in women or pharmacologic treatment, (4) systolic/diastolic pressure (SBP/DBP) ≥ 130/85 mmHg or antihypertensive drug treatment, and (5) fasting glucose (FAG) level ≥ 100 mg/dL or pharmacologic treatment.

### Epigenome-wide association study and meta-analysis for MetS

The KARE and HEXA studies utilized the Infinium MethylationEPIC BeadChip platform (850 K). The workflow of this study is shown in Additional file 1: Fig. S1. At baseline, methylation data were available for 865,918 CpGs in 1528 KARE samples and for 865,918 CpGs in 822 HEXA samples. Quality control of methylation data was conducted using the R package ChAMP [16] based on the following exclusion criteria: (1) probes with a detection p value above 0.01, (2) probes with fewer than three beads in at least 5% of samples per probe, (3) all non-CpG probes, (4) all SNP-related probes, (5) all multihit probes, (6) all probes located in chromosomes X and Y, and (7) samples without phenotype information. Based on these criteria, 724,619 CpG sites in 1,526 KARE samples (651 cases, 875 controls) and 732,046 CpG sites in 808 HEXA samples (163 cases, 645 controls) were included in the study.

For quality control, BMIQ [17] was used for normalization, and batch effects were corrected using a ComBat method [18] in the ChAMP package. Cell type compositions (CD8 T lymphocytes, CD4 T lymphocytes, natural killer cells, B lymphocytes, neutrophils and monocytes) were estimated using GLINT [19] with the ReFACTor algorithm [20].

We first identified CpGs for MetS and individual MetS components using Model 1 with a basic set of covariates (age, sex, residential area and estimated cell type compositions) using the KARE and HEXA datasets. Model 2 further adjusted for smoking status in addition to those covariates in Model 1. Model 3 included additional adjustment for body mass index (BMI) in Model 2. Furthermore, we performed stepwise regression analysis using the Akaike information criterion (AIC) to select the main covariates influencing DNA methylation changes in MetS (Additional file 3: Table S2).

To investigate the distribution of *p* values of test statistics, we used quantile–quantile (QQ) plots of observed and expected distributions of *p* values for each cohort. For more accurate statistical assessment, we estimated genomic inflation factors for each cohort and meta-analysis, using both the conventional approach and the *bacon* method [21], applied for a Bayesian method based on an empirical null distribution.

To improve statistical power through meta-analysis across different datasets at the individual CpG, we used bacon-adjusted cohort-specific results and conducted the inverse-variance weighted random-effects model, which was implemented in the ‘metagen’ function in the meta package (version 4.18.0). This approach allowed us to obtain a combined estimate of the effect size for DMP (differentially methylated probes) associated with a stringent threshold using Bonferroni correction (*p* < 7 × 10^−8^).

Differentially methylated regions (DMRs) can offer greater insight into biologically relevant DNA methylation changes [22]. Associations between diseases and DNA methylation are frequently observed in clusters of CpG sites located within specific DMRs. This observation aligns with the function of DNA methylation, as it can either enhance or inhibit the binding of transcription factors, thereby influencing gene expression [23]. Therefore, we performed a DMR analysis to investigate the joint effect of DNA methylation in whole blood on MetS and its components. For region-based meta-analysis, we used the comb-p [24] approach for detecting and testing DMRs enriched by multiple CpGs exhibiting the same direction of effects. The selected regions were defined based on the following criteria: a minimum of three CpGs within a region, a seed *p* value of less than 0.001, and a bin size of 310. To correct for multiple comparisons, we used a 5% Sidak corrected p value for significance [24]. The identified DMPs and DMRs were annotated using the Illumina UCSC (hg19 RefSeq gene annotation) and GREAT (Genomic Regions Enrichment of Annotations Tool) [25]. We also investigated regulatory regions for 15 different types of chromatin states, such as enhancers, using ChromHmm annotation to produce a universal chromatin state annotation of the human genome from the Roadmap Epigenomics and ENCODE projects [26]. Since our DNA methylation data were generated in whole blood samples, we chose peripheral blood mononuclear cells (PBMCs) as the reference epigenome E062 [27].

### Gene set enrichment analysis

We performed an enrichment analysis separately for DMPs and DMRs to facilitate the biological interpretation of methylation data obtained from the results of the meta-analysis. We identified the promoter region [within ± 2 kb around the transcription start sites (TSS)] based on the genes associated with specific regions and CpG sites. We performed functional annotations for the alterations observed in significant DMPs and DMRs via gene set enrichment analysis using the GREAT algorithm [25]. This analysis employs a hypergeometric test on MSigDB using Hallmark and Gene Ontology (GO) gene sets, with a significance threshold of an adjusted *p* value < 0.05.

### Genome-wide association study for MetS

The KARE and HEXA cohorts were genotyped with Affymetrix Genome-Wide Human SNP Array 5.0 and 6.0, respectively. The genotype data were available for 352,228 SNPs in 8840 KARE samples and 627,659 SNPs in 3693 HEXA samples. We used PLINK version 1.9 [28] for quality control with the following exclusion criteria: (1) SNPs on chromosomes X, Y, and mitochondria, (2) SNPs with a missing call rate greater than 5%, (3) SNPs with minor allele frequency below 5%, (4) SNPs with a Hardy‒Weinberg equilibrium p value of 1 × 10^−6^, and (5) samples without phenotype information. For subsequent analysis, reference genome annotation was converted from NCBI build 36 to GRCh37 matching methylation data using the LiftOver tool. [29] A total of 305,544 SNPs for 5888 KARE samples and 538,413 SNPs for 3668 HEXA samples remained after quality control. We performed logistic regression analysis for binary traits of MetS and linear regression analysis for continuous traits for MetS components. We corrected for multiple comparisons using a Bonferroni correction in the meta-analysis.

### Mendelian randomization test for meQTLs as causal CpGs to MetS

We conducted meQTL analysis in the KARE dataset. After LD pruning, we identified DNA methylation sites that may be influenced by SNPs using both genotyping and methylation data. To determine meQTLs, linear regression analysis was performed using the R package matrix eQTL [30] with age, sex, residential area, smoke, and cell type compositions as covariates. We tested for cis-meQTLs, defined as SNP and CpG sites located within 1 Mb of each other. Among the SNP-CpG pairs, the lead SNP for each CpG was defined as the pair with the smallest *p* value. To test the significant differences between two groups, t tests or chi-square tests were used.

To evaluate whether CpGs (exposure) might have a causal effect on MetS and individual components (outcome), we conducted MR using CpGs having ≥ 3 independent FDR-corrected meQTLs as instrumental variables (IV) (Additional file 3: Table S3). The association results of meQTL and GWAS were used for two-sample MR analysis to estimate the causal effects of DNA methylation on MetS because this analysis, except MR‒Egger, has demonstrated its reliability in the context of large biobanks, even in cases of complete sample overlap [31]. In our study, we used inverse-variance weighted (IVW) and weighted median (WM) to investigate possible causal effects of DNA methylation. The R package MendelianRandomization [32] was used for this analysis. The heterogeneity *p* value was obtained from Q-statistics.

## Results

### Characteristics of participants

The baseline characteristics of the study participants are presented in Additional file 3: Table S4. We finally included 814 participants with MetS and 1520 controls. The cohorts in our study consisted of population-based participants recruited independently from diseases and health status. The mean age ranged from 40 to 78 years across disease statuses, and the proportion of men ranged from 53.1 to 64.8% for MetS cases and controls, respectively. We observed higher mean age, BMI and MetS component levels in MetS cases than in controls. There were significant differences in age, sex, BMI, and smoking status between MetS cases and controls.

### EWAS identified significantly methylated CpGs for MetS and its components

The results of each cohort for the main models are plotted in Additional file 2: Fig. S2 (7 outcomes). There were some inflations in the models. The amount of inflation estimated using the lambda (*λ*) inflation factor varied substantially across analyses, ranging from 0.98 to 1.66. After removing inflation and bias, the $\lambda$ values ranged from 0.98 to 1.14.

**Fig. 1:**
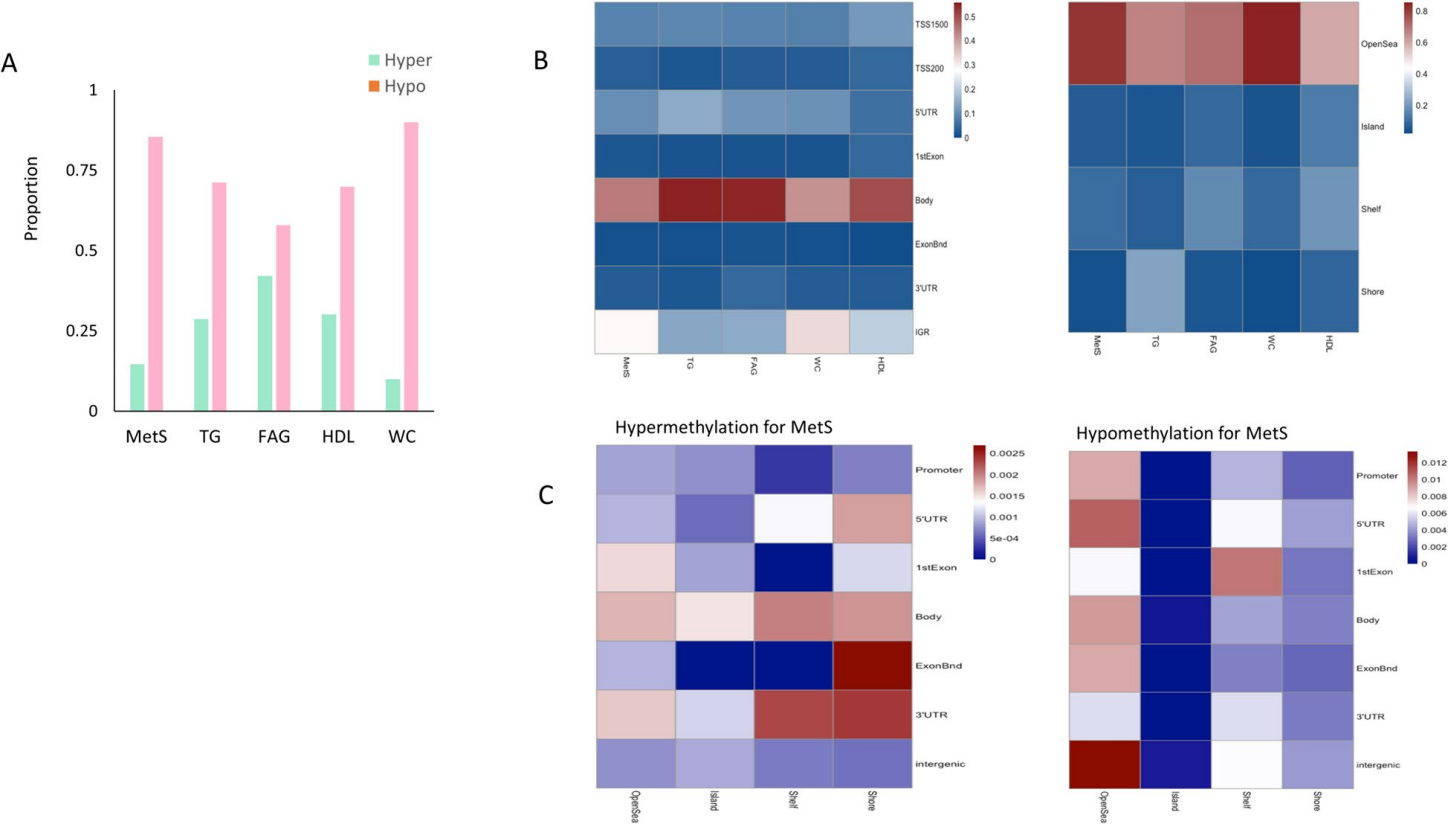
**A** Proportion of hypermethylation vs. hypomethylation in MetS and its components in meta-analysis. **B** Functional genomic distribution and neighborhood location of hyper and hypomethylated CpG sites in MetS and its components. The promoter region is located within 2 kb from the transcription start site. Intergenic regions are defined as the remainder of locations located between genes. **C** Distribution of genomic feature between hyper and hypo DMPs for MetS. Shores and shelves are composed of CpG methylation sites located 0–2 kb and 2–4 kb, respectively, from the nearest CpG Island; the open sea is defined as CpG methylation sites located > 4 kb from a CpG island. MetS, metabolic syndrome; TG, triglyceride; FAG, fasting glucose; HDL. High-density lipoprotein; WC, waist circumference

After correcting inflation for each cohort, we first annotated the less conservative CpGs (FDR < 0.05, Fig. 1) in the meta-analysis to examine the potential functional effect of DNA methylation on MetS by associating these CpGs with predetermined genomic features and regions. We observed a global genome-wide hypomethylation in MetS and its components (Fig. 1A). No significant CpGs were found in the DBP, and only two CpGs were found to be significant in SBP, all of which were hypomethylated. The genomic regions and features of DMPs are shown in Fig. 1B. The majority of the significant DMPs were located in gene body and open sea in MetS and all its components. In specific for MetS, there were different patterns of genomic regions and features between hyper and hypo DMPs. We found hyper DMPs were enriched in 3’UTR with shelf and shore, and ExonBand with shore (Fig. 1C, left). On the other hands, the majority of hypo DMPs were located in open sea and intergenic region (Fig. 1C, right).

**Fig. 2:**
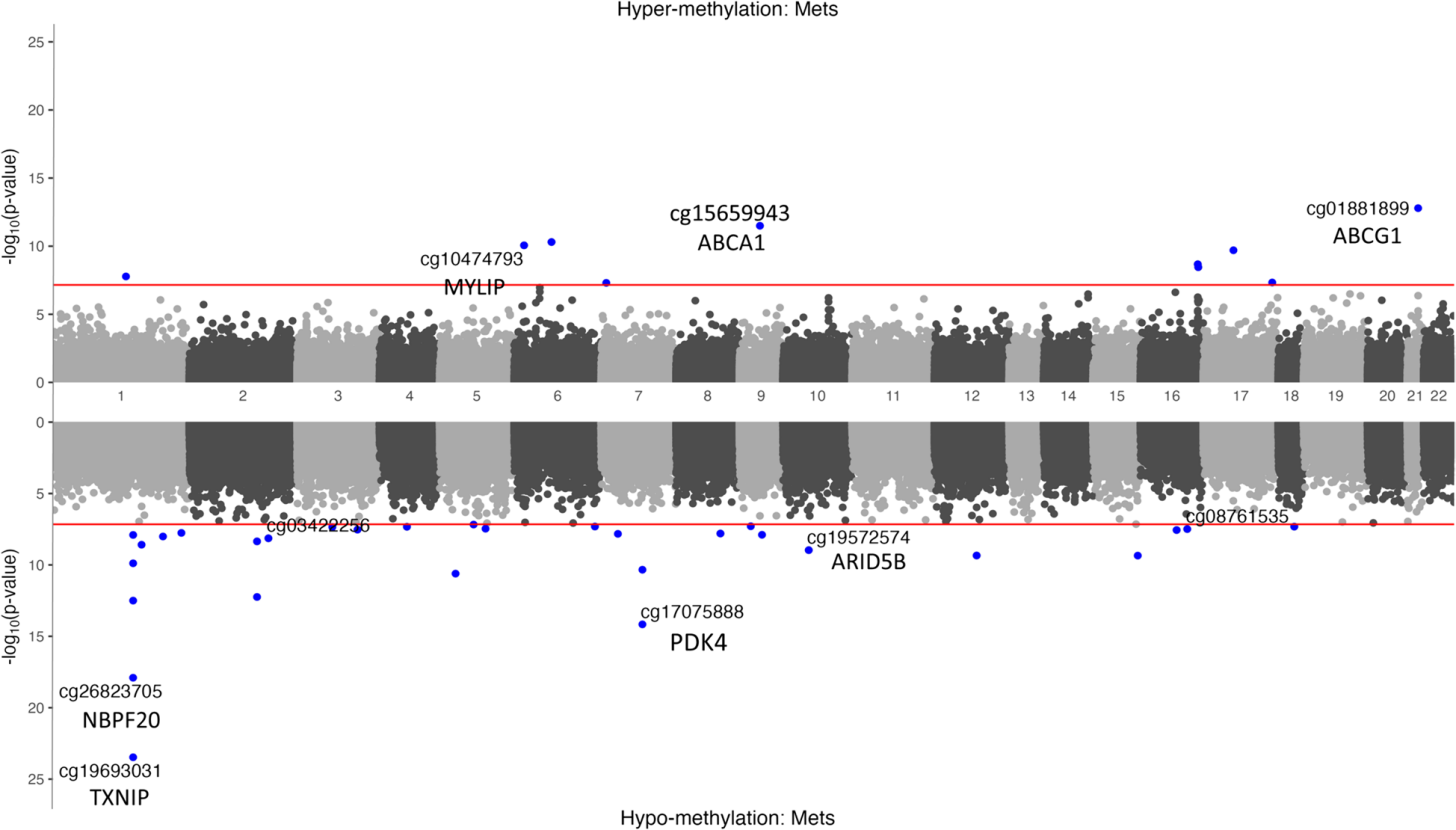
Miami plot using epigenome-wide meta-analysis for metabolic syndrome (*n* = 2334). The red line indicates the epigenome-wide significance level threshold to account for multiple testing (*p* < 7 × 10^−8^)

In the meta-analysis, we identified 40 bacon-corrected significant MetS-associated CpGs, of which 36 genes remained significant following Bonferroni correction for MetS (Table 1, Additional file 3: Table S5, Fig. 2). Among the 40 significant CpG sites related to MetS status, 30 (75%) were hypomethylated, while 10 (25%) were hypermethylated. Eight CpGs were located in CpG islands or the shore, while the remaining CpGs were located in the open sea. Only one of these 40 CpGs was located in the promoter of gene mapped to *RASSF9* and was hypomethylated. Interestingly, of the 40 CpG sites, 4 CpGs (cg10474793, cg08822075, cg01881899, and cg16734637) corresponded to enhancer regions and were mapped to the gene bodies of *MYLIP, NFE2L3, ABCG1* and *FOXP1*, respectively. The DMP gene *MYLIP* is associated with ligase activity, which regulates cholesterol uptake. *NFE2L3* is involved in ubiquitin protein ligase activity and transcription coactivator activity to bind antioxidant response elements. The *FOXP1* gene is associated with signaling pathways such as Wnt/Hedgehog/Notch.

**Table 1:** The top 20 significant differential methylated probes by epigenome-wide meta-analysis of whole blood methylation for metabolic syndrome

CpG	Chr	Position	Meta-analysis			Gene	Feature	Location	State	Dir	KARE			HEXA		
			Beta	SE	*P* value						Beta	SE	*P* value	Beta	SE	*P* value
cg19693031	1	145,441,552	− 11.43	1.13	3.39E−24	*TXNIP*	3'UTR	OpenSea	TxFlnk	–	− 11.17	1.38	4.77E−16	− 11.96	1.96	1.07E−09
cg26823705	1	145,435,523	− 13.50	1.53	1.26E−18	*NBPF20*	Body	OpenSea	TssAFlnk	–	− 14.02	1.86	5.46E−14	− 12.41	2.69	3.96E−06
cg17075888	7	95,225,339	− 9.64	1.24	6.88E−15	*PDK4*	Body	N_Shore	TssBiv	–	− 10.34	1.46	1.43E−12	− 7.85	2.33	7.72E−04
cg01881899	21	43,652,704	41.84	5.67	1.62E−13	*ABCG1*	Body	N_Shelf	Enh	+ +	43.24	7.25	2.40E−09	39.62	9.11	1.38E−05
cg02988288	1	145,440,445	− 32.80	3.24	3.17E−13	*TXNIP*	Body	OpenSea	TxFlnk	–	− 35.53	3.87	4.46E−20	− 26.45	5.91	7.48E−06
cg07995927	2	161,995,135	− 12.25	1.70	5.71E−13	*TANK*	5'UTR	OpenSea	TssAFlnk	–	− 12.88	2.10	8.59E−10	− 11.06	2.90	1.34E−04
cg15659943	9	107,631,656	15.82	2.27	3.20E−12	*ABCA1*	Body	OpenSea	TxWk	+ +	17.16	2.74	3.68E−10	12.87	4.06	1.52E−03
cg14180330	5	40,224,461	− 10.80	1.62	2.46E−11	*LINC00604*	IGR	OpenSea	Quies	–	− 11.78	1.97	2.12E−09	− 8.74	2.84	2.08E−03
cg00980461	7	95,238,409	− 13.07	1.99	4.60E−11	*AC002451.3*	IGR	OpenSea	Quies	–	− 12.50	2.44	3.05E−07	− 14.18	3.41	3.22E−05
cg07068382	6	36,947,277	14.93	2.27	4.97E−11	*MTCH1*	Body	OpenSea	Tx	+ +	14.80	2.71	4.63E−08	15.23	4.17	2.61E−04
cg10474793	6	16,134,001	13.22	2.04	8.70E−11	*MYLIP*	Body	OpenSea	EnhG	+ +	14.20	2.36	1.79E−09	10.35	4.04	1.03E−02
cg26974062	1	145,440,734	− 26.11	2.81	1.66E−10	*TXNIP*	Body	OpenSea	TxFlnk	–	− 28.31	3.29	7.21E−18	− 20.10	5.43	2.17E−04
cg25425189	17	37,349,801	15.86	2.49	2.00E−10	*CACNB1*	Body	N_Shelf	Tx	+ +	15.39	3.09	6.38E−07	16.72	4.22	7.29E−05
cg09880921	15	99,214,666	− 10.78	1.73	4.49E−10	*IGF1R*	Body	OpenSea	TxWk	–	− 10.68	2.08	2.89E−07	− 11.01	3.11	3.91E−04
cg27035734	12	86,230,557	− 13.84	2.22	4.56E−10	*RASSF9*	TSS1500	OpenSea	Quies	–	− 14.61	2.70	6.01E−08	− 12.22	3.91	1.79E−03
cg19572574	10	63,592,731	− 10.81	1.77	1.09E−09	*ARID5B*	IGR	OpenSea	Quies	–	− 11.52	2.13	6.22E−08	− 9.21	3.21	4.12E−03
cg16470089	16	87,782,144	8.83	1.47	2.12E−09	*KLHDC4*	Body	OpenSea	Tx	+ +	8.72	1.74	5.37E−07	9.10	2.78	1.04E−03
cg21575225	1	156,073,485	− 14.43	2.42	2.60E−09	*LMNA*	5'UTR	OpenSea	RepPCW	–	− 15.66	2.95	1.09E−07	− 11.87	4.25	5.25E−03
cg04583842	16	88,103,117	12.10	2.05	3.52E−09	*BANP*	Body	S_Shore	Tx	+ +	12.09	2.43	6.80E−07	12.13	3.80	1.40E−03
cg00021659	2	161,996,385	− 14.40	2.45	4.45E−09	*TANK*	5'UTR	OpenSea	TssAFlnk	–	− 14.91	3.10	1.53E−06	− 13.54	4.02	7.45E−04

Position on Hg19. Inverse-variance weighted random-effects meta-analysis models were used to combine dataset-specific results from logistic regression models that included covariate variables age, sex, regional area, smoking status, batch, and cell-type proportions. Direction (Dir) indicates hypermethylation or hypomethylation in blood compared to control. Genes are annotated by illumine and UCSC and GREAT annotation. TxFlnk, transcription at gene 5′ and 3′; TssAFlnk, flanking active transcription start site; TssBiv, bivalent/poised transcription start site; Enh, enhancer; TxWk, weak transcription; Quies, quiescent/low; Tx, strong stranscription; RepPCW, weak repressed polycomb

The most significant probe was cg19693031 (*p* = 3.37 × ${10}^{24}$), which mapped to thioredoxin interacting protein (*TXNIP*), which was hypomethylated and located in the open sea on chromosome 1q21.1. This site has been widely reported to be hypomethylated in T2D [33, 34]. In addition, we identified other significant DMPs (cg02988288, cg26974062) in *TXNIP* associated with MetS and its components (*p* < 2.0 × ${10}^{10}$ for all). These sites were associated with the TxFlnk (transcription at gene 5′ and 3′) region, which was reported to have both promoter and enhancer signatures, or the TssAFlnk region, which represents active TSS-proximal promoter states (Table 1) [26]. An additional CpG site, cg26823705, within *NBPF20* was also located in 1q21.1 and negatively associated with MetS. For each MetS component, we identified 63 (41 hypomethylated) CpGs for TG, 47 (20 hypomethylated) CpGs for FAG, 12 (11 hypomethylated) CpGs for HDL, 9 (7 hypomethylated) CpGs for WC, and 2 (2 hypomethylated) CpGs for SBP (Additional file 3: Tables S6–S10). However, there were no significant DMPs for DBP.

### EWAS identified significant DMRs for MetS and its components and its association with DMPs

Using region-based meta-analysis *p* values for individual CpGs as input, we identified 27 DMRs associated with MetS status at the 5% Sidak *p* value after multiple comparison corrections (Table 2). The most significant DMR, which encompassed 3 probes, was located at *TXNIP* (chr1:145440444–145441553, 2.5 kb from TSS, *p* value = 2.35 × 10^−58^). Importantly, this DMR was associated with the same gene as the top DMPs. Ten DMRs (located in the *SCD, DOK3, TNFA1P8, TNF, CPT1A, OXT, TM4SF1, TCTEX1D4, IL5RA, and SFRP2* genes) were located in the promoter region. These DMR-associated genes were implicated in the immunological pathway, lipid metabolism, and signaling pathway. For example, the DMR gene *SCD* is associated with fatty acid (stearoyl-CoA) biosynthesis, while the DMR gene *DOK3* is involved in the *Ras* signaling pathway.

**Table 2:** The significant differential methylated regions by epigenome-wide meta-analysis for metabolic syndrome

DMR	N_probes	Genes (distance to TSS)	*P* value	Sidak *P* value
chr1:145440444–145441553	3	TXNIP(+ 2530)	1.27E−38	3.65E−32
chr16:87781335–87782145	5	KLHDC4(+ 17,815)	7.38E−21	5.26E−19
chr10:102107583–102107758	4	SCD(+ 790)	5.39E−13	2.21E−09
chr5:150466792–150466840	3	DOK3(+ 130)	1.90E−12	2.89E−08
chr5:176936562–176936893	5	TNIP1(− 5819)	2.02E−12	4.39E−09
chr5:118689954–118689975	3	TNFAIP8(− 44)	2.38E−12	8.52E−08
chr12:26424986–26425212	5	SSPN(+ 76,670)	1.25E−11	3.97E−08
chr15:29407791–29408146	4	NDNL2(+ 154,064)	3.70E−11	7.48E−08
chr3:18480241–18480707	5	SATB1(− 14,409)	5.49E−10	8.44E−07
chr8:42037965–42038196	3	PLAT(+ 27,161)	5.83E−10	1.81E−06
chr6:31543539–31543687	8	TNF(+ 269)	6.19E−10	3.01E−06
chr11:68607621–68607738	4	CPT1A(+ 1704)	9.83E−10	6.06E−06
chr1:45274031–45274056	3	TCTEX1D4(− 1087)	2.04E−09	6.09E−05
chr10:44757322–44757371	3	CXCL12(+ 123,150)	2.98E−09	4.44E−05
chr20:3051953–3052346	10	OXT(− 116)	3.73E−09	6.81E−06
chr17:79005385–79005663	4	BAIAP2(− 3438)	5.55E−09	1.43E−05
chr3:149094652–149094893	3	TM4SF1(+ 879)	5.84E−09	1.74E−05
chr13:50702409–50702915	6	DLEU1(+ 46,355)	1.12E−08	1.58E−05
chr6:106546539–106546825	6	PRDM1(+ 12,487)	1.18E−08	2.96E−05
chr3:18459837–18459999	3	SATB1(+ 6147)	1.79E−08	7.94E−05
chr10:4093709–4093926	4	KLF6(− 266,351)	1.97E−08	6.51E−05
chr5:142562352–142562570	3	NR3C1(+ 220,793)	2.68E−08	8.84E−05
chr3:3151739–3152039	3	IL5RA(+ 169)	4.16E−08	9.94E−05
chr4:154711511–154711630	5	SFRP2(− 1299)	4.64E−08	2.81E−04
chr10:14062053–14062218	3	FRMD4A(+ 310,747)	6.57E−08	2.86E−04
chr3:156807519–156807692	4	CCNL1(+ 70,396)	7.52E−08	3.12E−04
chr10:6187993–6188416	4	PFKFB3(− 56,689)	8.51E−08	1.44E−04

DMR, differential methylated region; N_probes, number of probes for DMR; +, upstream from transcription start site (TSS); −, downstream from transcription start site

**Fig. 3:**
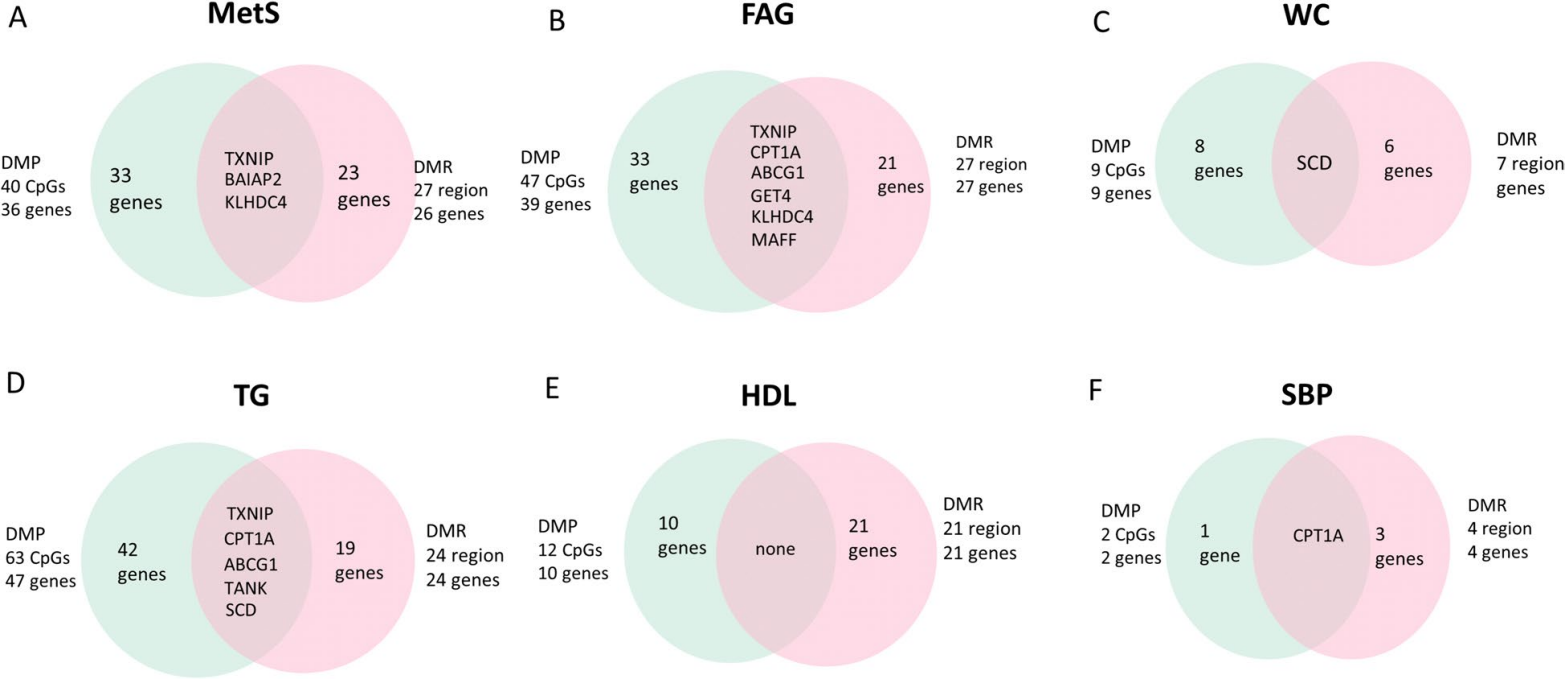
Venn diagrams showing overlap between genes corresponding to DMPs and genes corresponding to DMRs in each MetS and its components. The numbers of DMPs (green) and DMRs (pink) corresponding to genes are presented for each phenotype (**A**–**F**)

Furthermore, we analyzed intersections between genes corresponding to DMPs and genes corresponding to DMRs within MetS and each of its components individually (Fig. 3, Additional file 3: Table S11). The majority of the DMPs that overlapped with DMRs were limited to a single probe. The genes exhibiting an overlap between significant DMRs and DMPs for MetS were *TXNIP, BAIAP2* and *KLHDC4*; only five CpGs overlapped with DMR on these three genes. The significant DMPs and DMR overlapped within the *TXNIP* gene for FAG and TG as well as for MetS. We also observed that a significant DMP was in the DMR-associated gene *KLHDC4* (located at chr16:87781335–87782145), which is involved in intestinal inflammation in FAG as well as MetS [35]. Although there was no specific overlap, significant DMPs and DMRs coexisted within the *BAIAP2* gene*.*

**Fig. 4:**
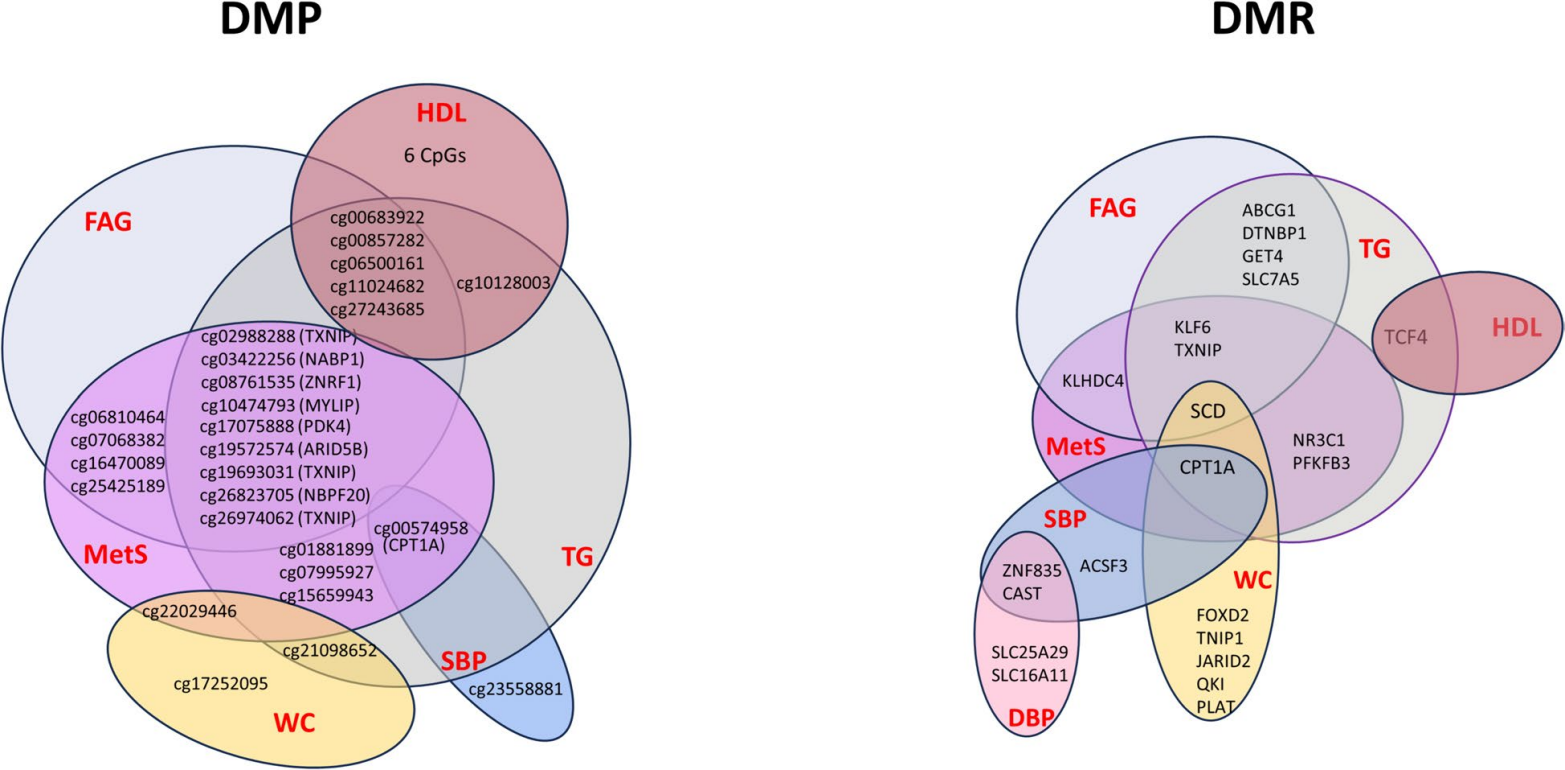
Venn diagrams showing overlap and unique DMPs and DMRs among MetS and its components. The gene TXNIP overlaps across MetS, TG and FAG in both DMPs and DMRs

Since MetS components are strongly interconnected with MetS [36], we investigated whether significantly methylated CpG sites in MetS are linked to other MetS components simultaneously. Therefore, we proceeded to explore the areas of overlap between MetS and its individual components within DMPs and DMRs separately (Fig. 4). Among the CpG sites associated with MetS, 18 CpG sites exhibited the most notable overlap with various MetS components. Of these, nine CpGs corresponding to 7 genes exhibited overlap in MetS, TG, and FAG. The site cg00574958 at *CPT1A* was significant in MetS, TG and SBP. Five CpG sites (cg00683922, cg00857282, cg06500130, cg11024682, and cg27243685) overlapped with the three MetS components, regardless of their direct association with MetS itself. For example, the cg06500161 site on *ABCG1* was observed in TG, FAG, and HDL, indicating that this CpG site overlapped with these three Mets components but not with MetS itself.

In the case of DMRs, the region with the highest degree of overlap between MetS components and MetS is located within *CPT1A,* which was differentially methylated in MetS, TG, WC, and FAG (Fig. 4). Additionally, *KLF6* and *SCD* also overlapped among MetS, TG, and FAG. Similar to DMPs, the most significant DMR-associated gene in each phenotype tends to overlap with both MetS and its individual components.

**Fig. 5:**
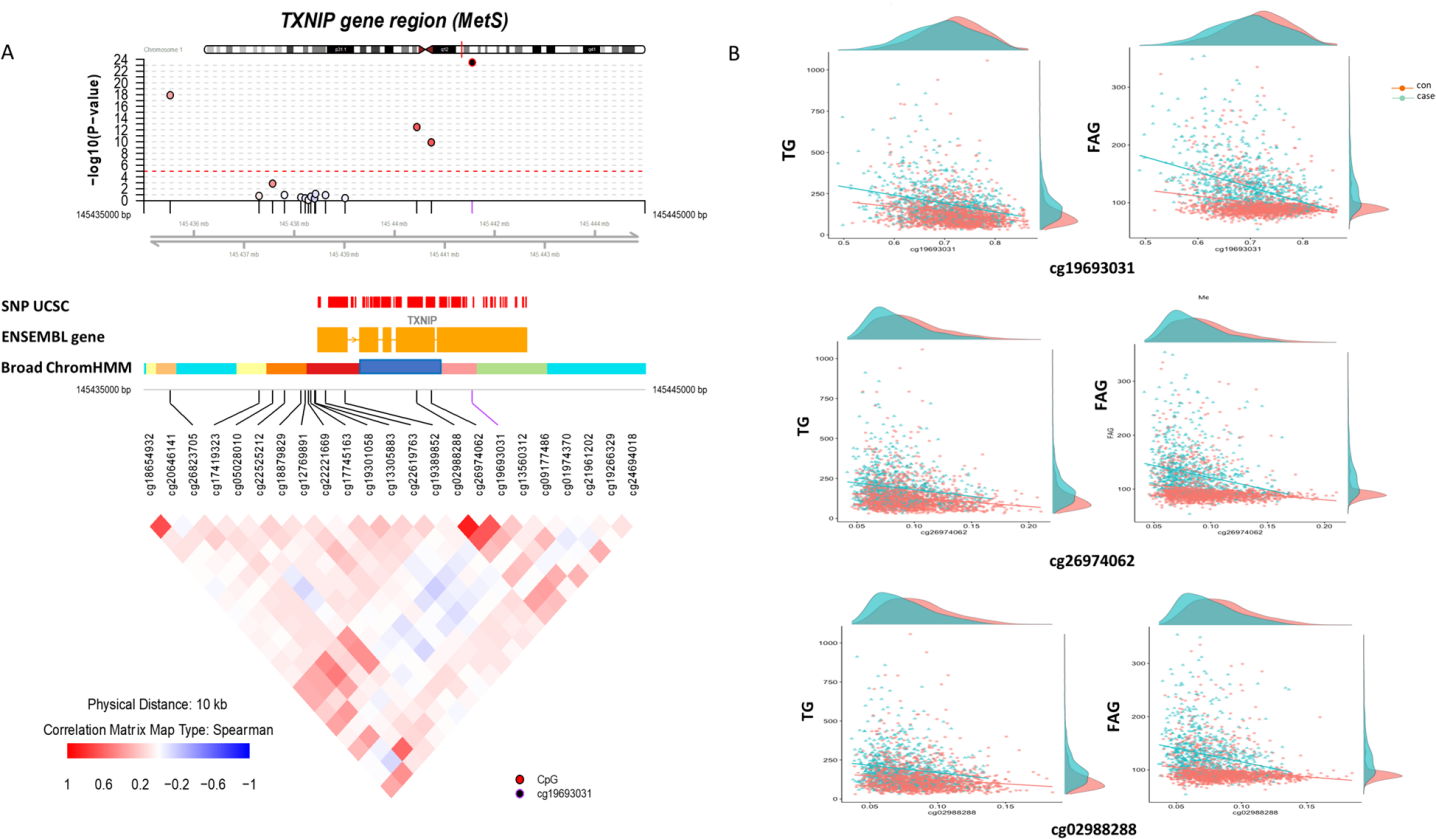
Metabolic syndrome-associated DNA methylation signals in *TXNIP*. **A** coMET plot describing the genomic region of association between *TXNIP* methylation and metabolic syndrome (top panel), along with functional annotation of the region (middle panel), and the pattern of comethylation at 23 CpG sites of *TXNIP* (bottom panel). **B** Correlation of DNA methylation at differentially methylated probes with TG and FAG

Neighboring CpGs have a higher likelihood of exhibiting similar methylated patterns. Therefore, we conducted a more detailed examination of the comethylation patterns by elucidating the genomic regions around the predominant CpG sites using the coMET plot (Fig. 5A). Visualization of regional DNA comethylation patterns showed that the most significant CpGs around the *TXNIP* gene had similar patterns in the same direction. Based on ENCODE data extracted from the UCSC GenomeBrowser (GRCh37/hg19 assembly), we found that the *TXNIP* sites overlapped with at least one crucial regulatory element, suggesting their location within a weak promoter region (Fig. 5A, pink in Broad ChromHMM). These regions are likely to play a role in transcriptional activity. In addition, we identified a nearly identical correlation pattern associated with three CpGs of *TXNIP* in the TG and FAG, as well as MetS (Fig. 5B).

### Gene set enrichment analysis for MetS by DMPs and DMRs

**Fig. 6:**
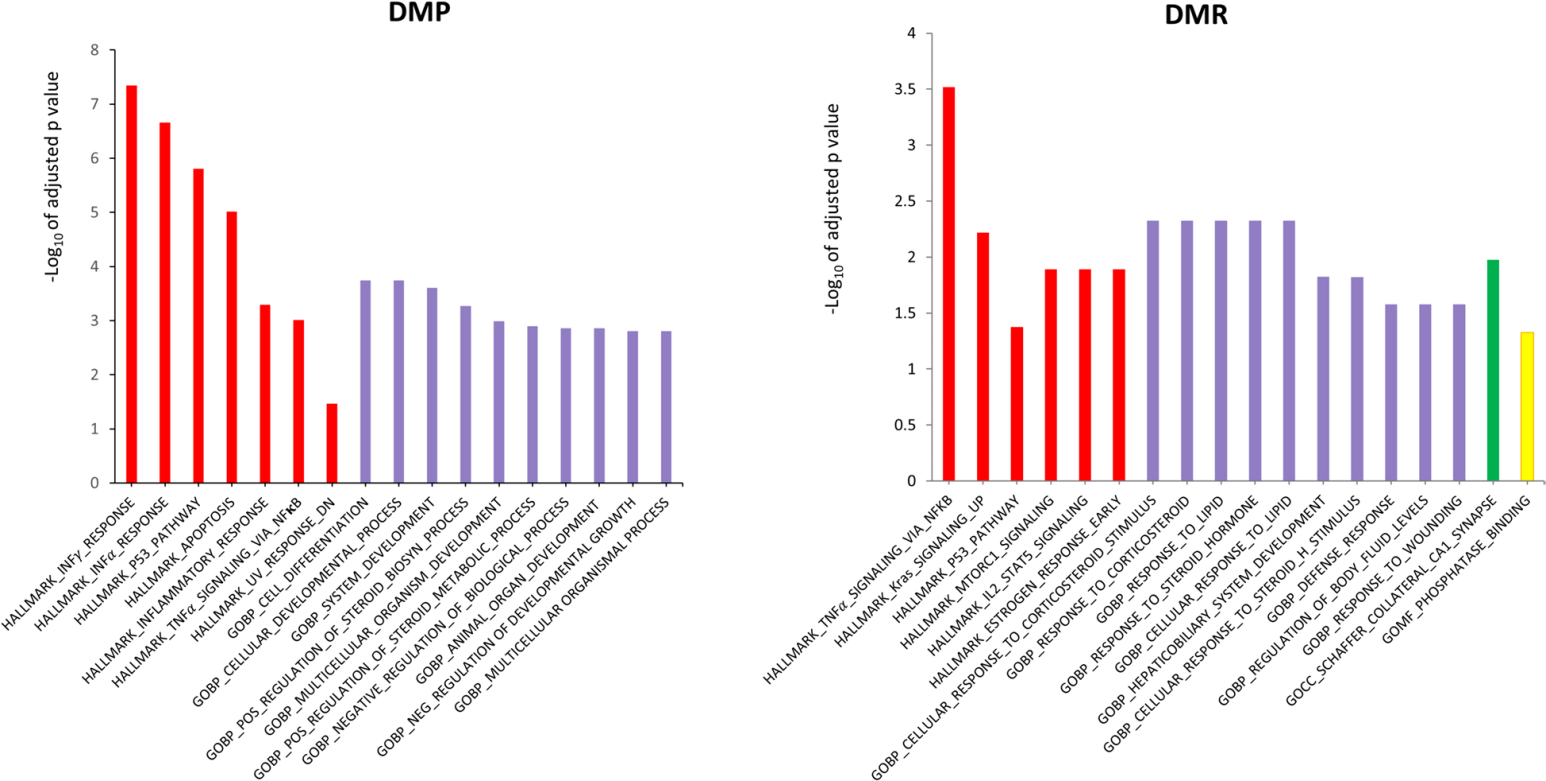
Gene set enrichment analysis using hallmark and gene ontology (GO) gene set database for DMP and DMR. DMP, differentially methylated probes for metabolic syndrome; DMR, differentially methylated regions for metabolic syndrome. The red bar includes the hallmark gene sets that represent key biological processes. GO Ontology categories genes by Biological Process (BP, purple), Cellular Component (CC, green), and Molecular Function (MF, yellow). P value is adjusted for hypergeometic test

To explore the biological processes influenced by DNA methylation, we conducted a gene set enrichment analysis using the Molecular Signature DataBase (MSigDB) Hallmark and GO gene set, employing meta-analyzed differentially methylated genes. As a result, we identified a high overlap between DMPs and DMRs associated with MetS, which induced differential methylation enriched at genes involved in inflammatory pathways, including TNF/IFN/NF-KB/STAT (tumor necrosis factor/interferon/signal transducers and activators of transcription) signaling pathways, as identified in the hallmark supersets. Additionally, the GO biological pathways illustrated glucocorticoid metabolic processes and steroid metabolic processes (Fig. 6) through both DMPs and DMRs. Glucocorticoids are steroid hormones synthesized by the adrenal cortex and play a crucial role in regulating a wide range of metabolic and homeostatic functions [37]. Collectively, these results suggest that the MetS state is associated with alterations in inflammatory function and hormone responses. Full tables of hallmark and GO pathway results can be found in Additional file 3: Tables S12 and S13.

### meQTL and MR analysis

Methylation quantitative trait loci, known as meQTLs, may represent specific genetic variants that respond to DNA methylation changes, thus potentially influencing MetS states. To investigate whether SNPs might be responsible for driving the methylation differences observed in MetS, we performed further analyses to identify meQTLs that influence the methylation levels of significant CpGs (Additional file 3: Table S14). To perform MR, we first checked the three assumptions for MR for causal estimates using two-sample MR. The results are summarized in Additional file 3: Table S3. Under the assumption of selecting the IV, we detected 3,212 cis-mQTLs. After FDR correction, we identified 144 cis-meQTLs that were associated with DNA methylation at 13 of the 40 DMPs related to MetS (Additional file 3: Table S14). The MR analysis identified 2 putative causal CpGs, cg01881899 at *ABCG1* and cg00021659 at *TANK,* from meQTL-CpG pairs for MetS with multiple testing correction (P_MR_ < 0.05/13, Table 3), and there was no evidence of heterogeneity among the genetic instruments (*p* for Q-statistics). The causal CpGs were negatively associated with MetS.

**Table 3:** Mendelian randomization of metabolic syndrome using cis-meQTL

CpGs	Gene	N of IV	IVW			WM			Cochran-Q	*P*_hetero_
			Estimate	se	*P*_ivw_	Estimate	se	*P*_wm_		
cg08357961	DISC1	15	− 3.67	1.65	0.03	− 1.05	2.39	0.66	13.68	0.47
cg08269908	ADGRG3	5	2.36	− 3.83	0.54	2.36	4.61	0.61	0.04	0.99
cg17075888	PDK4	36	− 2.82	0.77	2.59E − 04	− 2.45	1.02	0.02	5.96	0.99
cg09880921	IGF1R	7	− 7.83	4.28	0.07	− 9.85	4.18	0.02	18.61	< 0.001
cg00980461	AC002451.3	26	− 4.91	1.35	2.85E − 04	− 4.28	1.92	0.03	10.60	0.01
cg01881899	ABCG1	7	− 7.28	11.45	8.37E − 10	− 7.61	6.96	1.30E − 05	0.61	0.99
cg27035734	RASSF9	57	− 1.28	0.49	0.01	− 0.60	0.59	0.30	65.49	0.18
cg21623127	LIX1	6	− 8.05	3.04	0.01	− 8.08	3.80	0.03	0.02	0.99
cg00021659	TANK	41	− 9.11	1.21	2.10E − 07	− 8.10	1.58	3.52E − 07	13.49	0.99
cg00073751	LGR6	25	1.823	2.02	0.37	3.41	2.58	0.19	6.02	0.99
cg24562906	RNY4P18	6	3.344	3.54	0.34	2.40	4.39	0.58	1.04	0.96
cg08822075	NFE2L3	8	2.082	3.03	0.56	2.89	3.45	0.40	10.36	0.17
cg08761535	ZNRF1	6	1.362	3.42	0.69	2.23	4.12	0.59	0.56	0.99

IV, inference variables; IVW, inverse variable weight; WM, weight-median; For CpGs that tested causal for MetS and its components are shown in this table

## Discussion

It is crucial to identify the methylation sites or regions that play a causal role in MetS and elucidate how they interact with its component traits. This is vital for better understanding the underlying disease mechanisms for MetS, particularly in the context of complex diseases. In this study, we conducted EWAS on whole blood cells from 1526 MetS cases and 808 controls in a Korean population. As results, we found numerous DMPs and DMRs, which involved in the inflammatory response and lipid metabolism related to MetS. In particular, the presence of significant DMPs and DMRs that overlap with different components of MetS strongly suggests that they may collectively contribute to the manifestation of the disease. Furthermore, we were able to identify causal CpG sites influenced by genetic factors through MR.

It is highlighted that decreased DNA methylation at the top signals, specifically cg19693031, cg0298828, and cg26974062 in *TXNIP*, was associated with MetS status with elevated levels of FAG and TG. This association was further supported by significant DMRs observed for the same phenotypes. The CpG site with the strongest association (cg19693031) with MetS has been extensively studied and has shown links to blood lipid levels [38, 39], blood pressure [40], central obesity [41], and the prevalence of T2D and type 1 diabetes [33, 42, 43].

The hypomethylation of *TXNIP* (cg19693031) has previously been reported to be associated with MetS in Japanese and European populations. [6, 8] Similarly, other DNA methylation sites, including cg26974062 and cg02988828, have shown associations with maternal hyperglycemia and T2D as cg19693031 [44]. In contrast to cg19693031, the relationship between cg26974062 and cg02988828 and their association with these conditions has not been extensively studied or firmly established. Tobi et al. [44] commented that these two CpG sites are unique probes in the EPIC array. TXNIP serves as a pivotal regulator of glucose and lipid metabolism through multifunctional roles, such as modulating ꞵ-cell function, hepatic gluconeogenesis, peripheral glucose uptake, and adipocyte differentiation [45, 46]. Moreover, TXNIP plays a role across a broad spectrum of multiple cellular processes, including proliferation, differentiation, apoptosis, metabolism, and inflammation [34]. A significant finding in our study is the observation that three distinct DMPs located within a specific region of *TXNIP* collectively exert an influence on both MetS and its components, particularly FAG and TG levels. Notably, we confirmed 3 significant DMPs of TXNIP with DMR result. Based on these results, it can be speculated that DNA methylation, including the three CpG sites within TXNIP, exerts an influence on FAG and TG levels, thus playing a crucial role in the epigenetic processes involved in the development of MetS.

In general, studies with large sample sizes (> 1000) have estimated that approximately 10% to as much as 45% of the methylome is influenced by nearby meQTLs [11, 47]. MR provided evidence of a causal effect of DNA methylation associated with lead SNPs on multiple phenotypes. Therefore, pairwise association analyses were performed for target meQTL-24K SNP pairs and significant CpGs related to MetS and its components, as measured in blood samples from participants. Finally, we identified 144 meQTLs, among which only 13 CpGs (having > 3 meQTLs) were considered suitable for testing the causality of DNA methylation on MetS. After adjusting for multiple testing, the MR analysis finally identified two CpGs (cg01881899 of *ABCG1* and cg00021659 of *TANK*) as potential causal factors influenced by genetic effect for MetS. While cg01881899 did not align with the significant CpG sites of the *ABCG1* DMR, both DMPs and DMRs of *ABCG1* were linked to TG and FAG levels (Fig. 4).

Our study identified that cg01881899 of *ABCG1* is a CpG site influenced by 7 meQTL for MetS (Table 3), and it is also associated with TG [48], BMI [49] and HOMA-IR [50]. However, there has been limited research on the association between meQTLs and these phenotypes. We also found that the CpG site cg00021659 of *TANK* was influenced by 41 meQTL on MetS in this study (Table 3). We identified that cg00021659 is located in the 5’UTR and TssAFlnk state. TRAF family member-associated NF-kB activator (TANK) is a negative regulator of I-kappaB kinase/NF-kappaB signaling. A recent review has implicated the transcription factor NF-κB in the development of metabolic disorders, such as obesity, type 2 diabetes, and atherosclerosis [51]. However, it is a de novo CpG site that has not previously been reported to be associated with MetS and its components. It is known that genetic variants can influence a proportion of human DNA methylome. Our results may help establish causal relationships between genetic variants and DNA methylation and help to elucidate the underlying mechanisms of MetS.

There are some limitations with our study. First, we have DNA methylation data generated in whole blood, which includes multiple cell components. DNA methylation patterns are highly cell type- or tissue-specific, reflecting different methylation patterns that can vary depending on physiological conditions or disease states [52]. Although we controlled for cellular heterogeneity as a covariate, the putative effects of DNA methylation for MetS and its components in whole blood might not be identical to those occurring in target tissues involved in metabolic dysfunctions (i.e., adipose tissue, liver, etc.). Secondly, we need to ensure the reproducibility of the studies included in our meta-analysis to provide objective conclusions, which was challenging due to the lack of ethically matched samples. Future studies should be undertaken on MetS with a large number of samples as replication cohorts for facilitating cumulative scientific knowledge. Nevertheless, the discovery of changes of DNA methylation with regulatory state and meQTLs which can be essential for gene expression for MetS, may contribute the advancement of our understanding of the molecular pathways and help toward the development of therapeutic targets.

## Conclusion

We present meta-analysis involving MetS, highlighting numerous DMPs and DMRs in whole blood which are involved in inflammatory pathway, glucocorticoid metabolic processes and steroid metabolic processes. Especially 3 DMPs (cg19693031, cg26974062, cg02988288) and DMR (chr1:145440444–145441553) at *TXNIP* were significantly changed and warrant further study to explore their role in disease etiology. In addition, we identified a total of 144 cis-meQTLs, out of which only 13 were found to be associated with DMPs for MetS. Our results provide a comprehensive understanding of DMPs and DMRs in MetS and its components, as well as specific genetic effects on DNA methylation, subsequently affecting MetS status. We expect that these findings will be of use to the scientific community for further studies of epigenome regulation for MetS, and they may contribute to future research for prevention of MetS through epigenetic mechanisms.

### Supplementary Information

**Additional file 1**. Supplementary Figure 1.

**Additional file 2**. Supplementary Figure 2.

**Additional file 3**. Supplementary Tables 1–14.

## Data Availability

The Korean Genome and Epidemiology Study (KoGES) Consortium datasets in the current study are third party data and are available under the approval of the data access committee of the National Biobank of Korea, who can be contacted at http://biobank.nih.go.kr (82-1661-9070).
