# Supplementary figures and images for: Genome- and epigenome-wide association studies identify susceptibility of CpG sites and regions for metabolic syndrome in a Korean population
Source: Clin Epigenetics. 2024 Apr 29;16:60. doi: 10.1186/s13148-024-01671-5 (PMC11059751; doi:10.1186/s13148-024-01671-5)

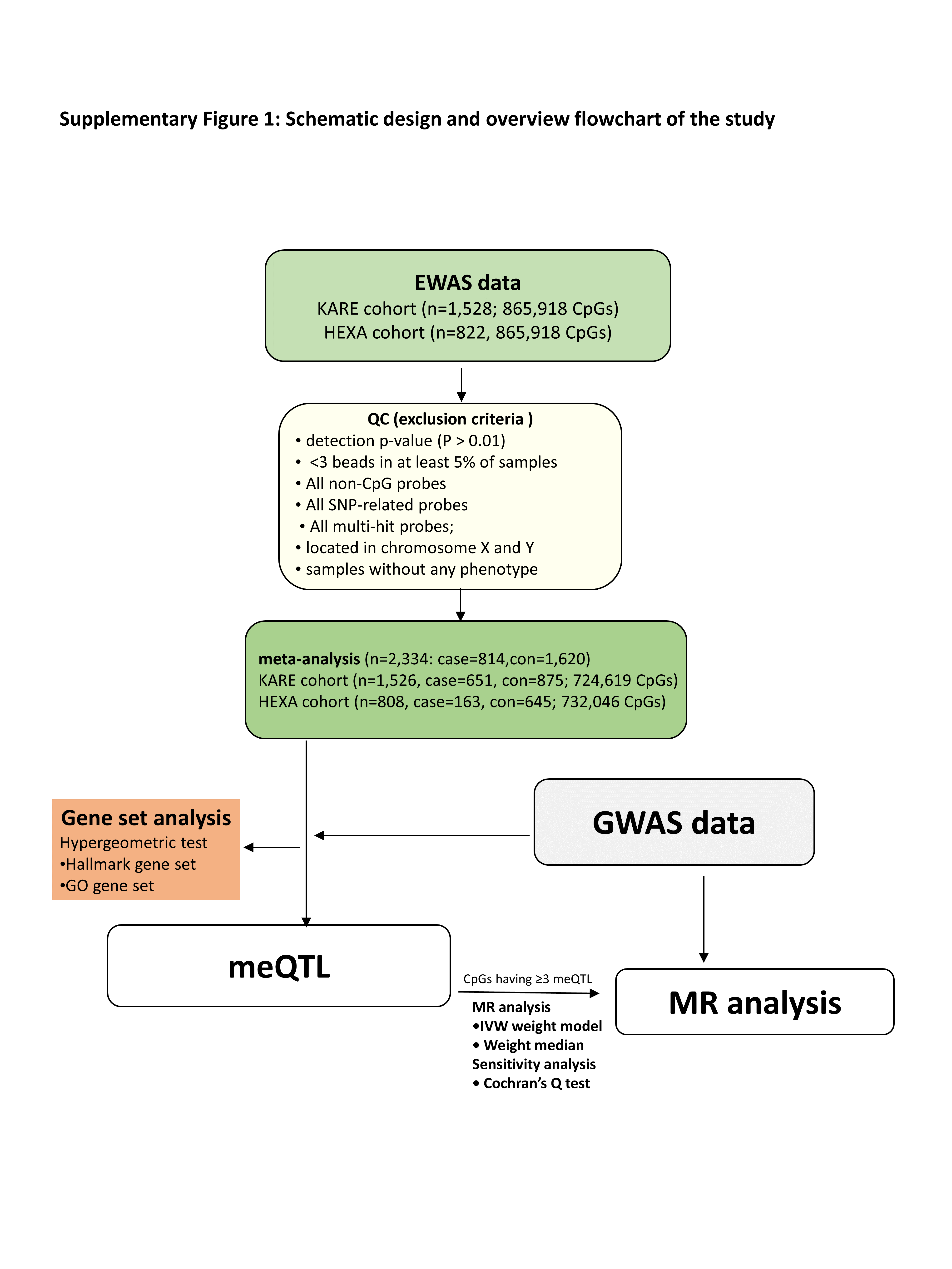

Supplement: Supplementary file 1 — Additional file 1. Supplementary Figure 1. [file 13148_2024_1671_MOESM1_ESM.png]
