# Supplementary material for: Genome- and epigenome-wide association studies identify susceptibility of CpG sites and regions for metabolic syndrome in a Korean population
Source: Clin Epigenetics. 2024 Apr 29;16:60. doi: 10.1186/s13148-024-01671-5 (PMC11059751; doi:10.1186/s13148-024-01671-5)
Supplement: Supplementary file 2 — Additional file 2. Supplementary Figure 2. [file 13148_2024_1671_MOESM2_ESM.pdf]

Supplementary Figure 2: Inflated epigenome-wide association study. Quantile-Quantile(QQ) plot of meta analysis data (KARE, n=1,526 and HEXA, n=808) by EWAS for MetS and its components. QQ plot show the observed minus log10-transformed P values obtained from a linear model corrected for biological and technical covariates against quantiles from the theoretical null distribution. P values in the –log10 scale for the GWAS meta-analysis**.** The shaded area indicated 99% concentration band. Results for KARE are indicated in *olive*, bacon-corrected KARE in *blue*, HEXA in *orange* and bacon-corrected HEXA in *green*.

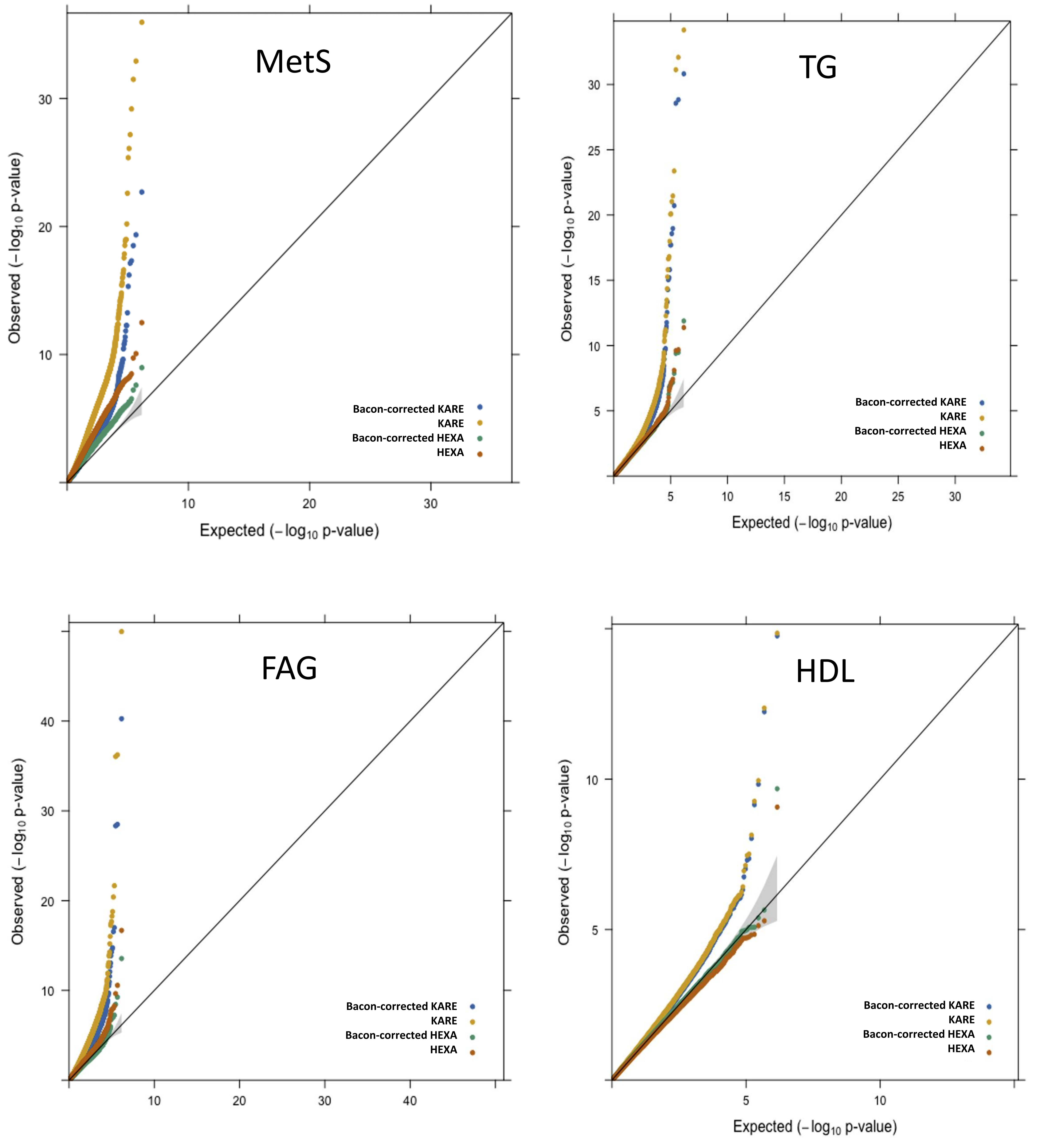

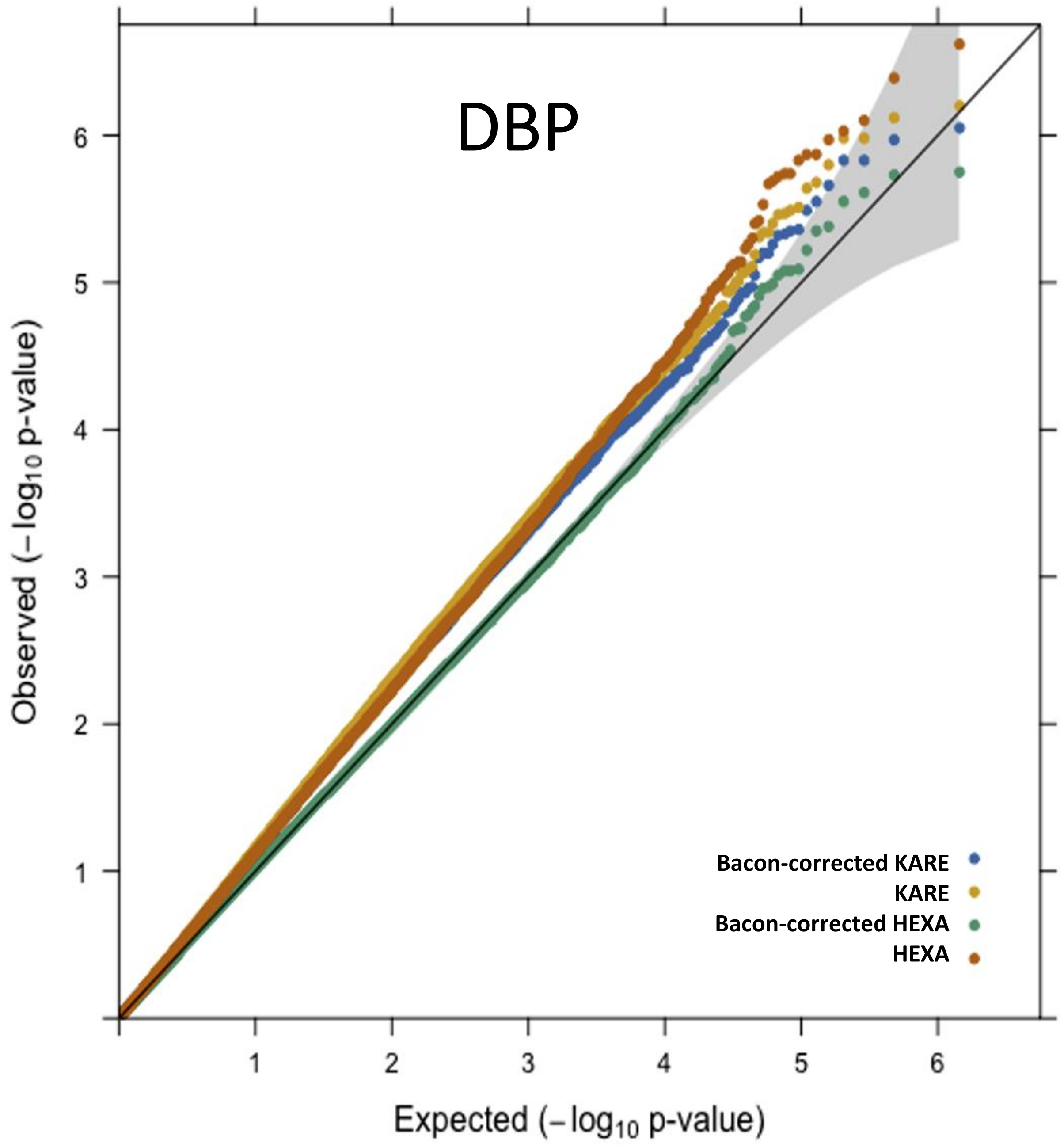
DBP
Observed (−log10 p-value)
Expected (−log10 p-value)
Bacon-corrected KARE
KARE
Bacon-corrected HEXA
HEXA

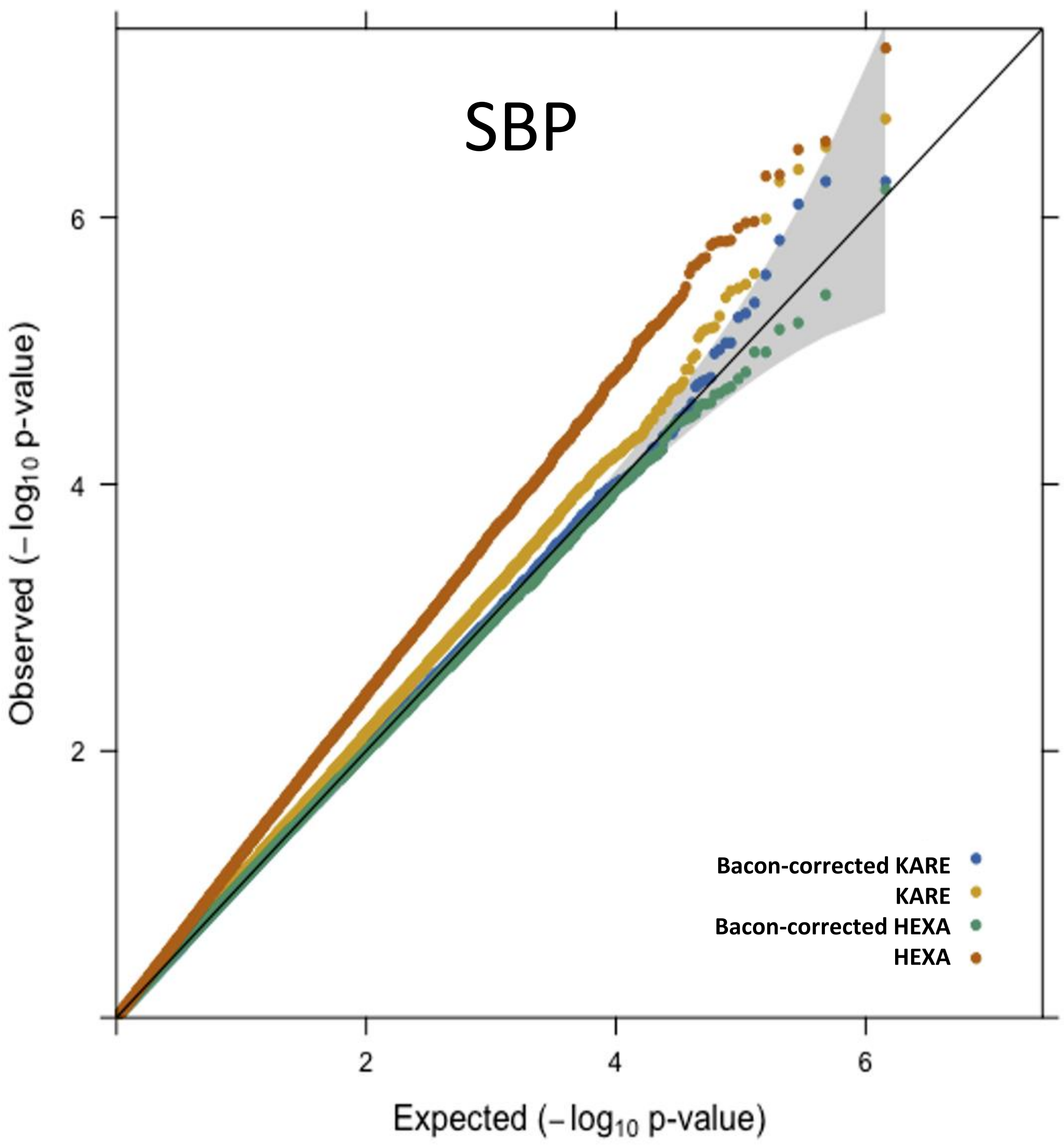
SBP
Observed (−log10 p-value)
Expected (−log10 p-value)
Bacon-corrected KARE
KARE
Bacon-corrected HEXA
HEXA

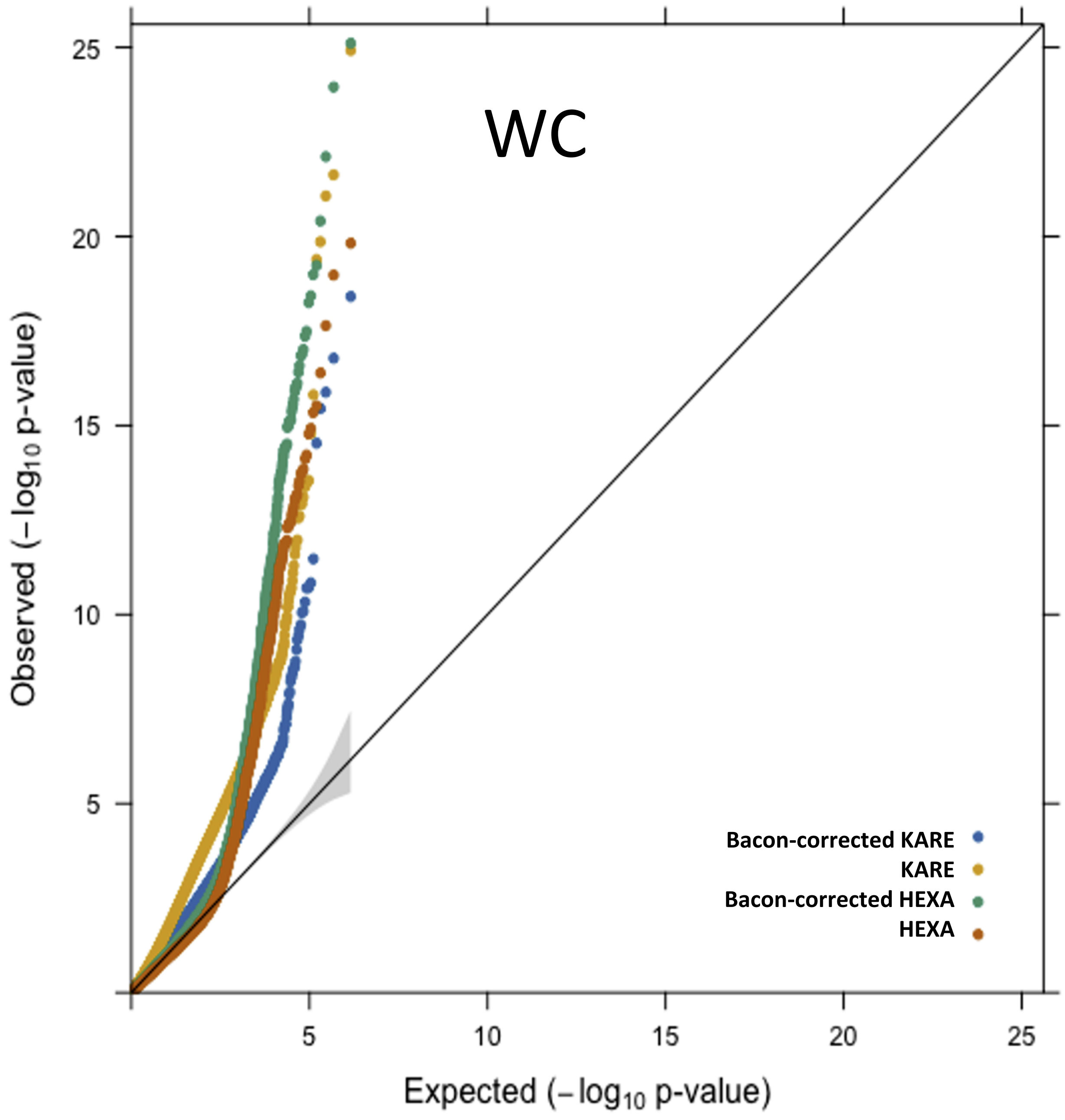
WC
Observed (−log10 p-value)
Expected (−log10 p-value)
Bacon-corrected KARE
KARE
Bacon-corrected HEXA
HEXA
